# Gene Set Enrichment Analysis and Genetic Experiment Reveal Changes in Cell Signaling Pathways Induced by α-Synuclein Overexpression

**DOI:** 10.3390/biomedicines11020263

**Published:** 2023-01-18

**Authors:** Yusong Huang, Dongjing Wen, Yao Yuan, Wenfeng Chen

**Affiliations:** Institute of Life Sciences, College of Biological Science and Engineering, Fuzhou University, Fuzhou 350108, China

**Keywords:** gene set enrichment analysis, Parkinson’s disease, α-Synuclein, signal pathway, *Drosophila*

## Abstract

Abnormal accumulation of alpha synuclein (α-Syn) in sporadic and familial Parkinson’s disease (PD) may be a key step in its pathogenesis. In this study, the expression matrix of the GSE95427 dataset after α-Syn overexpression in human glioma cell line H4 was obtained from the GEO database. We used the Gene Set Enrichment Analysis (GSEA) method to reanalyze this dataset to evaluate the possible functions of α-Syn. The results showed that the tumor necrosis factor alpha (TNF-α) signal was significantly activated in α-Syn-overexpressing cells, and oxidative phosphorylation signal, extracellular matrix signal, cell cycle related signal and fatty acid metabolism signal were significantly inhibited. Moreover, we employed the α-Syn-expressing transgenic *Drosophila* model of Parkinson’s disease and knocked-down eiger, a TNF superfamily ligand homologue, indicating that the TNF-α pathway plays a role in the common pathogenesis of synucleinopathies. Our analysis based on GSEA data provides more clues for a better understanding of α-Syn function.

## 1. Introduction

The protein α-Synuclein (α-Syn) was discovered in 1988 as a component of cholinergic synapses in the discharge organ of *Torpedo californica* [1]. In humans, α-Syn is abundant in the brain, primarily in synaptic terminals [2,3] at the tips of neurons. Pre-synaptic terminals transmit signals between neurons by releasing various neurotransmitters from synaptic vesicles, and are essential for normal brain function [4]. In 1997 and 1998, evidence that mutations in the α-Syn gene were found in sporadic and familial Parkinson’s disease (PD) [5,6]. This understanding of the pathogenesis of PD was considered by many scientists to be at least as important as the description of the toxicity of MPTP (a neurotoxin capable of destroying dopamine-producing nerve cells in the substantia nigra) 15 years ago [7]. The gene encoding α-Syn is also called “PARK1”, and its mutations, such as A30P and A53T, are closely related to PD. PD, the second most common neurodegenerative disorder, is characterized by a progressive loss of dopaminergic neurons within the substantia nigra pars compacta of the midbrain [8]. The neuropathological hallmark of the disease is the presence of intracytoplasmic inclusions called Lewy bodies (LBs) and Lewy neurites (LNs) [9]. Although it is clear that LB and LN mainly contain α-Syn, the mechanism leading to the aggregation of α-Syn needs to be clarified [10]. Perhaps α-Syn is a multidimensional factor that might subtend several neurobiological underpinnings. Thus, the cause of α-Syn aggregation and its relationship to dopamine neuron loss in PD is the subject of much current work [11,12,13]. The main problem in the molecular pathology of PD is not only to understand the aggregation of α-Syn, but also to know which key signals are associated with it, to regulate the pathological development of PD. Therefore, it is of great significance to study the gene networks related to α-Syn. However, many physiological functions of α-Syn are only partially understood; the exact pathomechanisms of α-Syn underlying neurodegenerative diseases remain elusive.

*Drosophila melanogaster* provides a simple, yet powerful genetic system for studying PD pathobiology in vivo. To date, various *Drosophila* models that mimic inherited forms of PD have been developed [14]. The power of *Drosophila* has revealed several genetic factors implicated in the various pathways of PD, and has given us a great understanding of the molecular mechanisms of dopaminergic (DA) neurodegeneration [14]. Although *Drosophila* has no homolog of the human Synuclein Alpha (SNCA) gene, pathogenic SNCA mutations that cause PD with a dominant inheritance pattern imply a hazardous gain-of-function mechanism, which leads to appropriate transgenic modeling in flies overexpressing wild-type or mutant α-Syn [15]. Using the usual Gal4/USA expression strategy, Feany and Bender first created α-Syn transgenic *Drosophila* models by overexpressing either wild-type or familial mutations A53T and A30P of human α-Syn [16]. These models replicate the key hallmarks of PD including adult-onset loss of DA neurons [15]. The α-Syn transgenic *Drosophila* models are widely used to identify novel proteins that cause α-Syn toxicity and to clarify underlying pathogenic processes [17].

In this study, according to the data published by Pinho et al. in 2019 [18], bioinformatics was used to reanalyze the related gene sets enriched after α-Syn overexpression to provide more clues and ideas for the study of α-Syn function. We used an α-Syn-expressing transgenic *Drosophila* model of PD to verify that the TNF-α pathway, one of the most obviously activated pathways shown by gene set enrichment analysis, is involved in the pathogenic processes of α-Syn.

## 2. Materials and Methods

### 2.1. Dataset Acquisition

As described previously by Pinho et al. [18], the GSE95427 dataset was obtained from GEO data (https://www.ncbi.nlm.nih.gov/geo/, accessed date: 10 May 2021) using α-Syn overexpression and gene chip analysis in human glioma cell H4. We obtained the expression matrix file (Series Martix File) with robust multiarray average (RMA) standardization processing and the corresponding probe platform information file (GPL570 [HG-U133_Plus_2] Affymetrix Human Genome U133 Plus 2.0 Array) [19].

### 2.2. Differential Expression Matrix Acquisition

We installed and loaded the Bioconductor package (https://bioconductor.org/, accessed date: 24 December 2018) in R3.5 software. Bioconductor was developed in the R statistical programming language, which is a popular toolkit for analyzing high-throughput genomic data [20]. After reading the original expression matrix we found that the platform information used by the GSE95427 dataset was GPL570, and its corresponding package file in Bioconductor was hgu133plus2.db. After installing the package, the corresponding relationship between the gene name information and the probe can be obtained, and then the probe and gene name can be converted to obtain the final expression matrix. Finally, the group matrix of the α-Syn overexpression group and control group was constructed using the limma package [21] in Bioconductor, and the differentially expressed genes were output after Bayesian test and linear model fitting.

### 2.3. Acquisition of Heat Map and Volcano Map

All differentially expressed genes were analyzed using the Subset function, and 806 genes with differences greater than twice and *p*-values less than 0.05 were found. The 806 differentially expressed genes were mapped using the Pheatmap function, and the heat map analysis results were obtained. All the obtained differential genes were plotted using the ggplot2 function to obtain a volcano map, and the names of the genes with a log2fold ratio change of more than 2 were displayed on the map.

### 2.4. GO and KEGG Analysis

The ClusterProfiler package [22] in the Bioconductor toolkit was used to obtain a gene list from 806 genes with a difference of more than twice and a *p* value of less than 0.05, and the gene names were converted into ENTREZID to represent genes in the NCBI database. GO analysis of cell components, biological processes, and molecular functions was performed using the function enrichGO, and KEGG [23] analysis was performed by the function enrichKEGG, with the partition *p*-value pvalueCutoff = 0.01 and the estimated false discovery rate qvalueCutoff = 0.01. Finally, the Barplot function was used to plot.

### 2.5. Gene set Enrichment Analysis

After loading the clusterProfiler package, regardless of the fold of gene differential expression, the gene list was obtained from all genes, the gene names were converted to ENTREZID, and the genes were sorted according to their differential expression values from high to low. The Hallmark gene set file was obtained from the MSigDB database [24], gene set enrichment analysis was carried out with GSEA function, and the overall distribution map was obtained using the dotplot function. After installing the enrichplot package (https://github.com/GuangchuangYu/enrichplot, accessed date: 24 December 2018), the gseaplot2 function in the package was used to obtain enrichment information maps of individual gene sets.

### 2.6. Drosophila Culture, qRT-PCR Analysis and Immunostaining of Fly Brains

Flies were routinely maintained in standard molasses-cornmeal-yeast food at 25 °C and raised at 29 °C for the experiments. w^1118^ (BS#5905), TH-Gal4 (BS#8848), UAS-αSynA30P (BS#8147), UAS-eiger-RNAi (BS#55276), and UAS-RFP (BS#32219) were obtained from Bloomington *Drosophila* Stock Center.

Head RNA from 25–30 days old flies (w^1118^, TH-Gal4 > UAS-RFP, TH-Gal4 > UAS-αSynA30P) was extracted using TRIGene (GenStar, P118-05). HiScript III RT SuperMix was used to generate the cDNA (Vazyme, R323). qPCR was carried out on a Roche LightCycler 96 using a RealStar Green Fast Mixture (GenStar, A301). The following mRNAs were quantified using primers designed using FlyPrimerBank (https://www.flyrnai.org/flyprimerbank, accessed date: 5 January 2023): actin5c-f: AGGCCAACCGTGAGAAGATG; actin5c-r: GGGGAAGGGCATAACCCTC; eiger-f: TATGACTGCCGAGACCCTCA; eiger-r: AAAACCAGGGGGATCAGCTG; Grnd-f: ATGGAGAGAGTAGGGATTGCC; Grnd-f: TGGGTTTGATTATTGCAGACCTC; Wgn-f: ACCATCTGCGGTTCCATATACG; Wgn-r: GTGCTCATACTCGGAGGACTT. The fold-change in expression relative to the control was calculated using the 2^−ΔΔCT^ method. Actin5c was used as an internal control.

Thirty days old flies (TH-Gal4 > w^1118^, TH-Gal4 > UAS-αSynA30P, TH-Gal4 > UAS-αSynA30P + UAS-eiger-RNAi) were fixed in 4% formaldehyde (Merck, Darmstadt, Germany, 8775) at 25 °C for 2 h. Brains were then dissected, washed in PBS with 0.2% Triton X-100 (PBST), and blocked in 5% goat serum in PBST for 1 h at room temperature. Primary rabbit anti-TH antibody (Merck, Darmstadt, Germany, AB152, 1:1000) was added and the samples were incubated overnight at 4 °C. Samples were washed three times with PBST, Alexa Fluor 488-labeled goat anti-rabbit secondary antibody (ThermoFisher, Waltham, MA, UAS, R37116, 1:50) was added, and the samples were incubated for 2 h at 25 °C. After washing with PBST and mounting, images were taken using a Leica TCS SP5 confocal microscope.

## 3. Results

### 3.1. Differentially Expressed Genes

**Figure 1:**
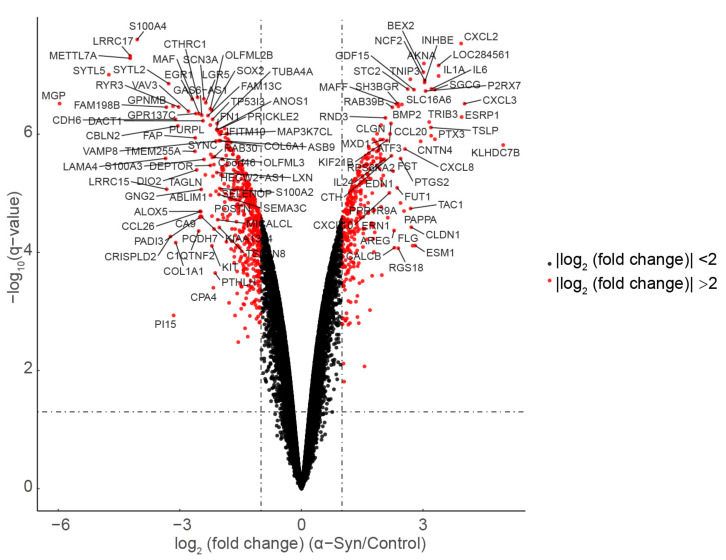
Volcano plot of differentially expressed genes.

To analyze differentially expressed genes in response to α-Syn overexpression it is necessary to construct a linear model, which is a common method for experimental data analysis. The limma package allows the construction of linear models and differential expression analysis. This package allows for simultaneously comparison of multiple experimental groups [21]. First, a linear model was fitted for each gene expression, and then Empirical Bayes was used to analyze the residuals to obtain the appropriate t-statistic, which was optimized for the variance estimation of the experiment to make the analysis results more reliable [21]. After constructing the matrix of the α-Syn overexpression group and control group using the limma program package, 20,186 of all differentially expressed genes were output after the steps of the Bayesian test and linear model fitting (Figure 1). Further, a total of 806 genes with a difference of more than twice and a *p* value of less than 0.05 were screened (Figure 1).

### 3.2. Clustering of Differential Expression Profiles

**Figure 2:**
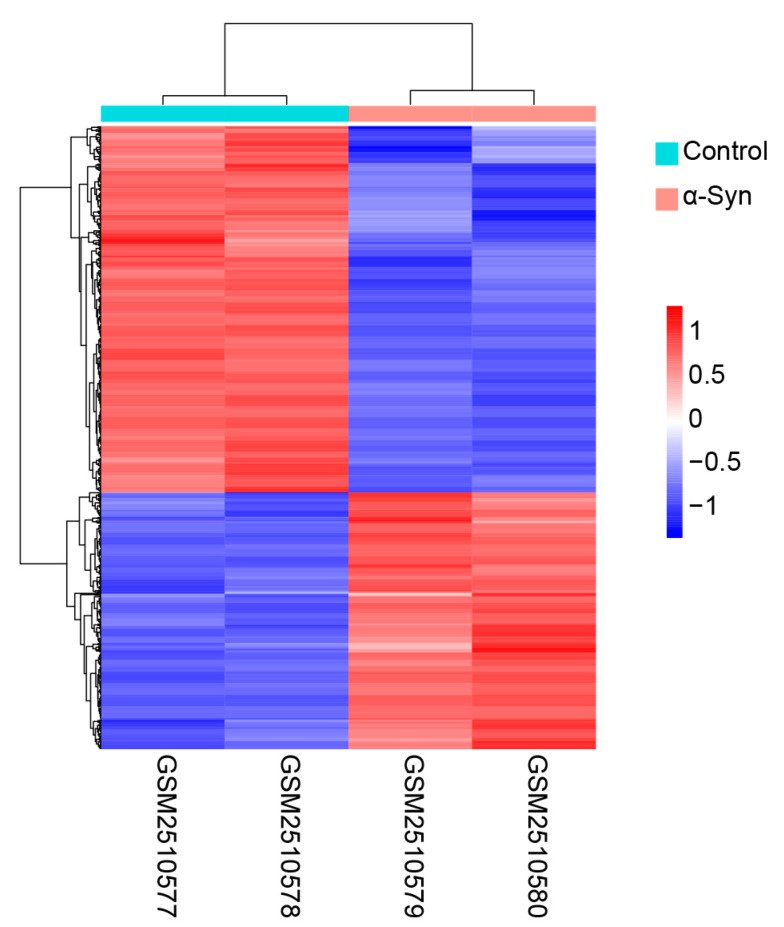
Cluster analysis of differentially expressed genes in α-Syn overexpressing H4 cells.

Clustering analysis was performed on 806 genes with a difference of more than twice and a *p*-value of less than 0.05. By observing the dendrogram (cluster analysis of rows and columns), it is evident that the expression patterns of the two samples belonging to the α-Syn overexpression group were similar, as were the two samples belonging to the control group (Figure 2). Compared to the control group, 332 genes were upregulated and 474 genes were downregulated in the α-Syn overexpression group (Figure 2). This indicates that both the control group and the α-Syn overexpression group had good reproducibility of their respective samples, which proves that there is no significant problem in the experimental treatment and that the data are reliable for further analysis.

### 3.3. Interaction Network of Differentially Expressed Genes

**Figure 3:**
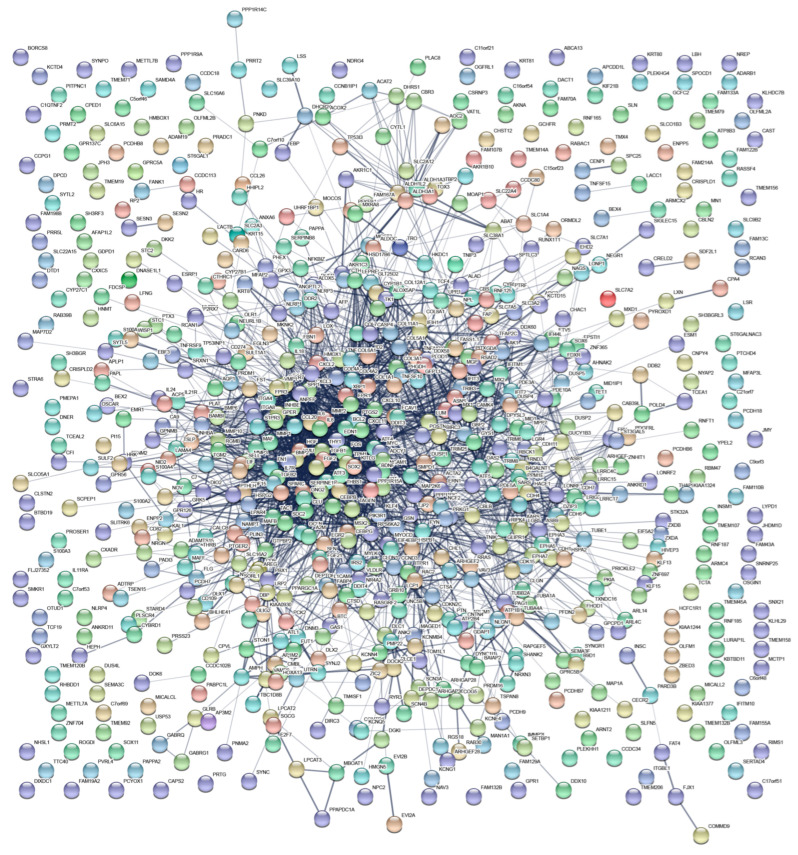
Proteins interaction networks for differential genes.

The STRING database (https://string-db.org/cgi/input.pl, accessed date: 10 May 2021) is a database for searching protein interaction on line [25]; it was searched, and the names of 806 genes with a difference of more than twice and a *p*-value less than 0.05 were used as inputs. Finally, the network view was used to show the direct or predicted association network of a specific protein. We found that 707 of the 806 differentially expressed genes could be densely located in a network through protein interactions, accounting for 87.7% of the total differentially expressed genes (Figure 3) and indicating that the genes with differences after α-Syn overexpression were very closely linked.

### 3.4. GO and KEGG Enrichment Analysis

**Figure 4:**
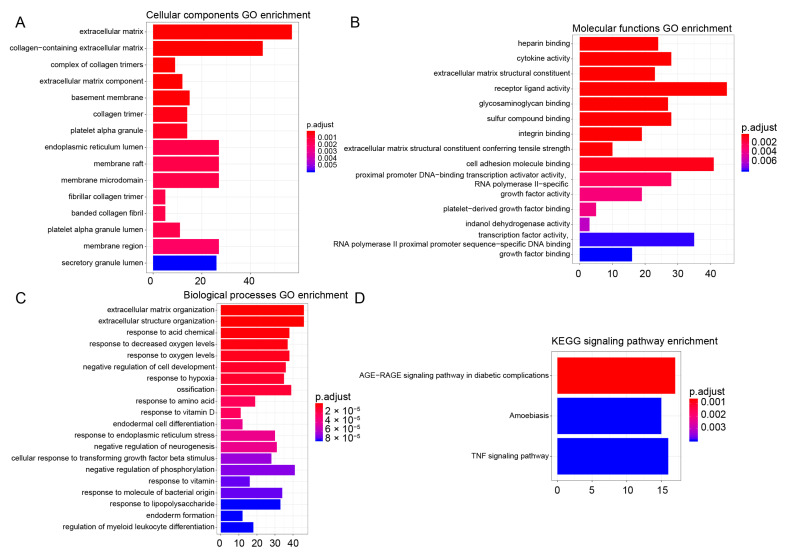
GO and KEGG enrichment analysis results. (**A**) GO enrichment of cellular components. (**B**) GO enrichment of molecular functions. (**C**) GO enrichment of biological processes. (**D**) KEGG signaling pathway enrichment.

The differentially expressed genes were analyzed by traditional Gene Ontology (GO) and Kyoto Encyclopedia of Genes and Genomes (KEGG) pathway enrichment analysis. GO enrichment analysis can be divided into three categories: cellular component, molecular function and biological process. According to the 806 differentially expressed genes selected above, the hypergeometric distribution relationship between these differentially expressed genes and some specific branches in the known GO classification was calculated. GO analysis will return a *p*-value for each GO with differential genes, and a small *p*-value indicates that the differential genes are enriched in GO. The results showed that extracellular matrix signals were significantly enriched in cellular components, molecular functions and biological processes after α-Syn overexpression (Figure 4A–C). In addition, KEGG pathway enrichment analysis revealed that only a few signals, such as TNF-α signaling pathway, was enriched after α-Syn overexpression (Figure 4D).

### 3.5. Gene Set Enrichment Analysis

**Figure 5:**
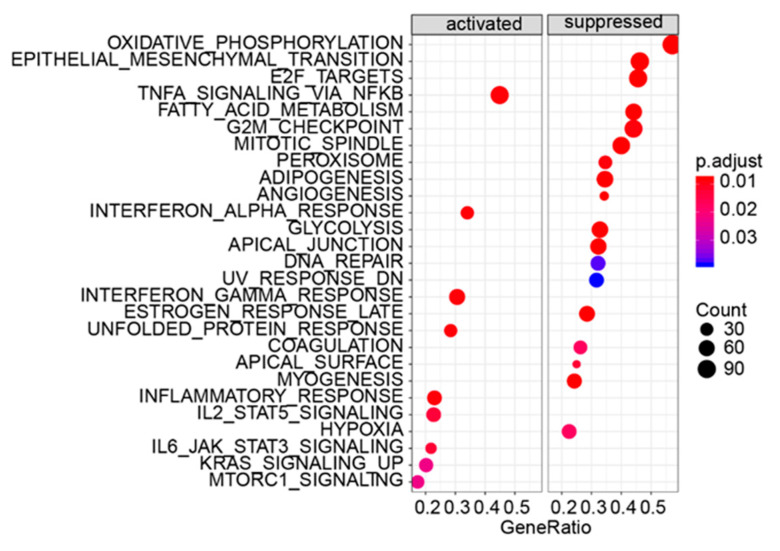
Gene set enrichment analysis results.

Given the limited amount of information obtained from the GO and KEGG enrichment analysis, gene set enrichment analysis (GSEA), a method that can examine the enrichment signal, was utilized to further analyze the data [24,26]. The GSEA method defines a specific gene set related to a particular biological process in advance based on existing research results, and then uses statistical methods to evaluate the distribution trend of all genes obtained in a specific experiment (regardless of the gene differential expression fold) corresponding to the predetermined gene set. Its input data consist of two parts: one is the preset known gene sets (which can be GO annotation, KEGG annotation, or other custom gene set conforming to the format), and the other is the expression matrix obtained by the experiment according to the change in gene expression value from large to small, and then judge whether the genes under each annotation in the gene set are enriched in a certain position of the expression matrix. GSEA analysis did not consider the fold of differential expression of genes, and the input variable was the differential expression of all genes in the two comparison groups, while the traditional GO and KEGG analysis methods artificially set the false discovery rate (FDR) to screen out a certain proportion of differentially expressed genes, and the input variable was a specific gene list. Therefore, compared with traditional GO and KEGG analysis, the results of GESA are more scientific. Here, GSEA was used to reanalyze the gene set enriched after α-Syn overexpression. The results showed that in the Hallmark gene set, overexpression of α-Syn activated 9 sub-gene sets and inhibited 18 sub-gene sets. The most obvious set of activating genes was the set associated with TNF-α signaling (NFKB-dependent TNF-α signaling) (Figure 5 and Table 1). However, oxidative phosphorylation-related signals (oxidative phosphorylation), extracellular matrix-related signals (epithelial-mesenchymal transition), and cell cycle-related signals (E2F targets) were significantly inhibited (Figure 5 and Table 1). The details of the genes involved in the top four enriched gene sets are shown in Table 1. Simultaneously, we found that the genes involved in the top four enriched gene sets appeared to different degrees in the protein interaction networks (Figure 3), especially for the TNF-α signal-related gene set and extracellular matrix-related signal: TNF-α signal-related gene set (39/88), oxidative phosphorylation-related signals (3/112), extracellular matrix-related signals (41/92), and cell cycle-related signals (4/89).

### 3.6. Reliability Analysis of Gene Enrichment

**Figure 6:**
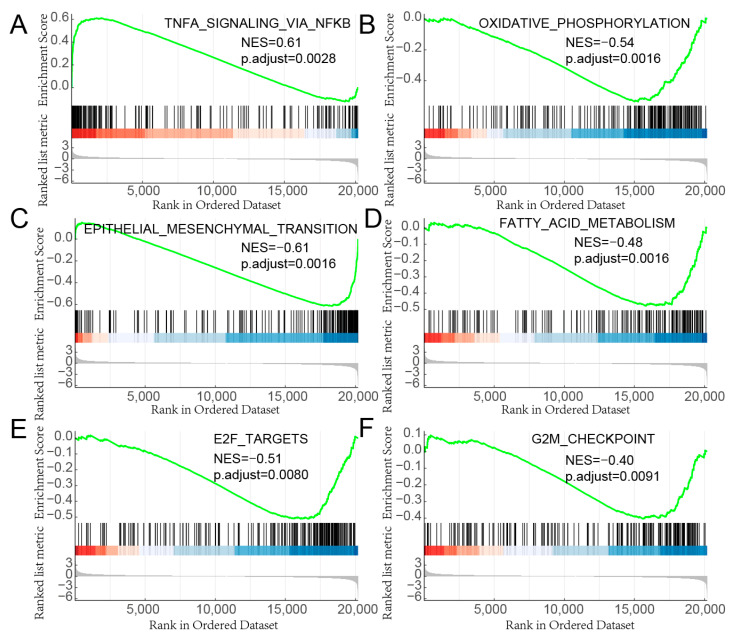
Details of single gene set enrichment. (**A**) Genes regulated by NF-kB in response to TNF-α. (**B**) Genes encoding the proteins involved in oxidative phosphorylation. (**C**) Genes defining epithelial-mesenchymal transition, such as in wound healing, fibrosis and metastasis. (**D**) Genes encoding proteins involved in fatty acids metabolism. (**E**) Genes encoding cell cycle-related targets of E2F transcription factors. (**F**) Genes involved in the G2/M checkpoint, as in progression through the cell division cycle. In the GSEA enrichment graphs, the curves represent the running sum of enrichment scores, the middle part of the graph shows the position of genes associated with specific pathways, and the bottom part of the graph shows how the fold change is distributed along with the gene list. The normalized enrichment score (NES) and the adjusted *p*-values were shown in the graphs.

To further evaluate the reliability of the results of the gene enrichment analysis, the gseaplot2 function was used to obtain the enrichment information map of a single gene set. We selected 6 gene sets to view detailed enrichment information. From the density distribution and enrichment scores of the ranking association matrix in the gene list it can be seen that both activated and repressed gene sets show relatively reliable enrichment, indicating that these gene sets are indeed significantly enriched (Figure 6).

### 3.7. Knockdown of TNF-α Pathway Partially Rescues the DA Neurons Degeneration Caused by α-Syn Overexpression

**Figure 7:**
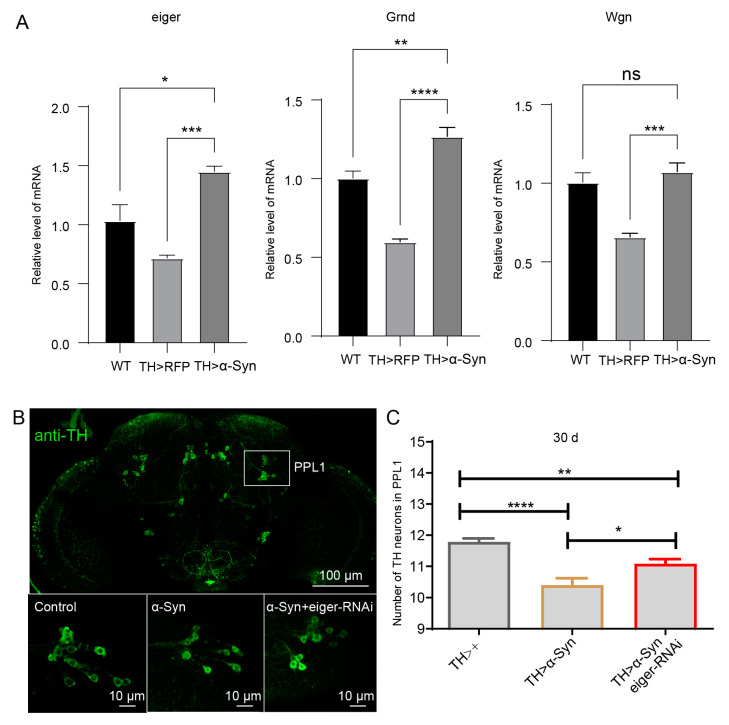
Eiger knockdown partially rescues the DA neurons degeneration caused by α-Syn overexpression. (**A**) Expression levels of eiger, Grnd and Wgn. (**B**) Images showing PPL1 neurons stained with anti-TH. (**C**) Quantification of PPL1 neurons number. One-way ANOVA analysis with Tukey’s multiple comparisons test was performed. * *p* < 0.05, ** *p* < 0.01, *** *p* < 0.001, **** *p* < 0.0001, ns no significance.

To further confirm the results obtained from GSEA we employed the α-Syn-expressing transgenic *Drosophila* model of PD for the in vivo experiment. The TNF-α pathway was chosen for further investigation because it was strongly activated in the top four enriched genes following α-Syn overexpression. We performed qRT-PCR detection of the *Drosophila* TNF-α homologue eiger [27] and its two receptors, Grindelwald (Grnd) [28] and Wengen (Wgn) [29]. Tyrosine hydroxylase (TH) is the rate-limiting enzyme for catecholamine synthesis, catalyzes the hydroxylation of tyrosine to dopamine [30], and is widely used to immunolabel DA neurons in *Drosophila* [31,32,33,34,35,36]. We found that α-Syn.A30P overexpression with a tyrosine hydroxylase promoter driver (TH-Gal4) [37] in dopamine neurons significantly increased the levels of eiger and Grnd in the head (Figure 7A). In *Drosophila*, we and other studies have shown that age-dependent specific loss of lateral protocerebral posterior 1 (PPL1) neurons was detected in several models of PD, including mutants for PINK1 [35], parkin [36], and α-Syn or Lrrk2 overexpression models [34,37]. When α-Syn.A30P was introduced into the DA neurons with TH-Gal4 driver, significant suppression of PPL1 DA neuronal loss was detected in the aged flies (Figure 7B,C). Interestingly, when the eiger was knocked down, it partially prevented PPL1 DA neurons degeneration in α-Syn.A30P overexpression flies (Figure 7B,C). Thus, we demonstrate that the TNF-α pathway is a physiological ligand in the α-Syn-mediated PD model.

## 4. Discussion

TNF-α is increasingly being recognized as a key pro-inflammatory cytokine involved in chronic inflammation and PD neurodegeneration. Microglial release and recombinant TNF-α have been shown to disrupt α-Syn degradation and lead to its accumulation in PC12 cells and midbrain neurons [38]. TNF-α can affect the autophagy pathway and regulate lysosomal acidification in dopaminergic cells, leading to the α-Syn accumulation. This may represent a novel mechanism for TNF-α-induced degeneration of dopaminergic neurons in PD. Another study also observed an increase in TNF-α expression after injecting α-Syn into the striatum of mice [39]. Both KEGG signaling pathway enrichment and gene set enrichment analyses showed that TNF-α-related signals or gene sets were significantly activated in cells overexpressing α-Syn, which is consistent with previous studies. Our study found that both eiger and Grnd were up-regulated in the α-Syn overexpression *Drosophila* heads. A recent study showed that Grnd and Wgn have different affinities for Eiger [40]. Ectopic Eiger expression leads to high-affinity interactions of Eiger: Grnd complexes in vesicles, which is a prerequisite for Eiger-induced apoptosis [40]. They also found that Wgn binds to Eiger with a much lower affinity in intracellular vesicles.

Studies have shown that the α-Syn protein can enter neurons and localize in mitochondria, interact with ATP synthase α subunit, and regulate the ATP synthase function [41]. However, this regulation appears to be opposite to that we found in the GSEA analysis. They found that low monomeric α-Syn had the ability to increase ATP synthase efficiency. As a physiological regulator of mitochondrial bioenergy, the α-Syn protein improves the efficiency of energy production through its interaction with ATP synthase. This may be particularly important when stress or PD mutations lead to energy depletion or neurotoxicity. In contrast, another study found that the highly neurotoxic α-Syn protein induces mitochondrial damage and mitochondrial autophagy [42], which is the site of oxidative phosphorylation. Gene set enrichment analysis revealed that oxidative phosphorylation was significantly inhibited. Therefore, how oxidative phosphorylation affects the aggregation of α-Syn protein and the pathological process of PD remains to be further studied.

Studies have also explored the role of cell cycle changes in neurodegenerative diseases such as Alzheimer’s disease. In one study, the authors examined the effect of α-Syn protein expression levels on the cell cycle index of PC12 cells and found that overexpression of α-Syn protein resulted in an increased rate of cell division, and a large number of cells appeared enriched in the S phase [43]. The α-Syn accelerates cell cycle and promotes neurotoxicity [44]. In addition, the research team from which the dataset was derived in this study also found that α-Syn causes severe transcriptional dysregulation, including downregulation of important cell cycle-related genes [18]. Gene set enrichment analysis revealed that several cell cycle-related gene sets were inhibited, such as E2F_TARGETS and G2M_CHECKPOINT, which provides more possibilities for future research on cell cycle changes, α-Syn protein aggregation and the impact on the pathological process of PD.

In addition to the above enriched gene sets, extracellular matrix signaling, DNA damage repair signaling, misfolded protein response, peroxisome-related signaling, inflammation, and immune signaling were significantly affected by α-Syn overexpression. Moreover, H4 cells are rather glial cells than neurons. The data obtained using H4 cells may be more specific to glial cells than to neurons. Multiple system atrophy (MSA) is another representative synucleinopathy [45]. It is well known that in MSA the α-Syn deposition is mainly observed in glia cells [46]. If the results using H4 cells are further examined in MSA models, more substantial conclusions may be obtained. Furthermore, lund human mesencephalic (LUHMES) cells can differentiate into neuronal cells with a robust dopaminergic phenotype [18]. In PD, major degeneration occurs in the dopaminergic neurons [47]. Analysis of the LUHMES-cell dataset may be more feasible for the study of PD. From the original point of view, the new factors found in the present study (Table 1) should be examined in vivo in future studies. If these new factors are validated in the future and translated into human medicine, they may be used as potential biomarkers or treatment targets for PD. In conclusion, the results of the gene set enrichment analysis provide more clues and ideas for future studies on α-Syn function.

## Figures and Tables

**Table 1:** The gene lists for the top four enrichment gene sets.

Gene Sets	Gene Symbol of Enrichment Genes in NCBI Database
TNF-α_SIGNALING_VIA_NFKB	AREG/ATF3/BCL2A1/BIRC3/BMP2/BTG1/CCL20/CD69/CD83/CEBPB/CEBPD/CSF2/CXCL10/CXC/CXCL2/CXCL3/DENND5A/DRAM1/DUSP1/DUSP4/DUSP5/EDN1/EIF1/FJX1/FOSL1/GADD45A/GCH/HBEGF/IER3/IFIH1/IFIT2/IL15RA/IL1A/IL6/IL6ST/INHBA/IRF1/IRS2/KDM6B/KLF4/KLF9/KYNU/LDLR/LIF/MAFF/MCL1/MSC/MXD1/MYC/NAMPT/NFIL3/NFKB1/NFKB2/NFKBIA/NFKBIE/NR4A2/OLR1/PHLDA1/PNRC1/PPP1R15A/PTGS2/PTX3/RCAN1/REL/RELA/RELB/RIGI/RIPK2/SAT1/SERPINB2/SERPINB8/SIK1/SLC16A6/SLC2A3/SOCS3/SOD2/SQSTM1/TGIF1/TIPARP/TNFAIP3/TNFAIP6/TNFRSF9/TNIP1/TRIB1/TSC22D1/VEGFA/YRDC/ZBTB10
OXIDATIVE_PHOSPHORYLATION	ACAA1/ACAA2/ACADM/ACADSB/ACADVL/ATP1B1/ATP5F1B/ATP5F1C/ATP5F1D/ATP5F1E /ATP5MC1/ATP5MC3/ATP5ME/ATP5MG/ATP5PB/ATP5PD/ATP5PF/ATP5PO/ATP6AP1/ATP6V0B/ATP6V0C/ATP6V0E1/ATP6V1D/ATP6V1E1/ATP6V1F/ATP6V1H/BAX/BDH2/CASP7/COX4I1/COX5A/COX5B/COX6A1/COX6B1/COX6C/COX7A2/COX7B/COX8A/CS/CYB5A/CYB5R3/DECR1/DLAT/ECH1/ECHS1/ETFA/ETFB/ETFDH/FDX1/FXN/GPI/GPX4/HSD17B10/HTRA2/IDH2/IDH3A/IDH3G ISCA1/LDHA/MDH1/MGST3/MPC1/MRPL15/MRPL34/MRPL35/MRPS11/MRPS12/MTRF1/NDUFA2/NDUFA3/NDUFA4/NDUFA5/NDUFA6/NDUFA7/NDUFA8/NDUFB1/NDUFB2/NDUFB3/NDUFB5/NDUFB6/NDUFB7/NDUFB8/NDUFS3/NDUFS6/NNT/OAT/OGDH/PDHB/PHYH/POLR2F/PRDX3/RETSAT/SDHB/SDHD/SLC25A11/SLC25A12/SLC25A20/SUCLA2/SUCLG1/TCIRG1/TIMM10/TIMM13/TIMM8B/TOMM22/UQCR10/UQCR11/UQCRB/UQCRC1/UQCRFS1/UQCRQ/VDAC1/VDAC3
EPITHELIAL_MESENCHYMAL_TRANSTION	ACTA2/ANPEP/APLP1/BASP1/BMP1/CALD1/CALU/CAP2/CAPG/CD59/CDH11/CDH6/COL11A1/COL12A1/COL16A1/COL1A1/COL3A1/COL4A1/COL4A2/COL5A1/COL7A1/CRLF1/CTHRC1/DCN/DPYSL3/ECM1/EDIL3/EMP3/FAP/FAS/FBLN5/FBN1/FBN2/FLNA/FN1/FSTL3/FUCA1/GAS1/GEM/GLIPR1/GPC1/HTRA1/ID2/IGFBP2/ITGA5/ITGAV/ITGB3/ITGB5/LAMA1/LAMA2/LAMC1/LGALS1/LOXL1/LRRC15/LUM/MATN2/MATN3/MGP/MMP2/MYLK/NID2/P3H1/PCOLCE/PDGFRB/PDLIM4/PLOD1/PLOD2/PLOD3/PMEPA1/PMP22/POSTN/PTHLH/RHOB/SDC1/SERPINE1/SFRP1/SGCB/SNAI2/SPARC/SPOCK1/SPP1/TAGLN/TGFB1/TGFBI/TGM2/THBS1/THY1/TIMP1/TNFRSF11B/TPM1/TPM4/VCAN
E2F_TARGETS	ASF1B/ATAD2/AURKA/BIRC5/BRMS1L/BUB1B/CBX5/CCNB2/CCNE1/CCP110/CDCA3/CDCA8/CDK1/CDK4/CDKN1A/CDKN2C/CDKN3/CENPE/CENPM/CKS2/DCLRE1B/DCTPP1/DLGAP5/DNMT1/DONSON/DSCC1/DUT/E2F8/EED/ESPL1/GINS1/GINS4/H2AX/HELLS/HMMR/JPT1/KIF22/KIF4A/KPNA2/LIG1/LMNB1/MAD2L1/MCM3/MCM4/MKI67/MSH2/MXD3/MYBL2/NCAPD2/NME1/PAICS/PCNA/PDS5B/PLK1/PLK4/PNN/POLA2/POLD1/POLD2/PPM1D/PRIM2/PRKDC/PRPS1/RACGAP1/RAD51AP1/RANBP1/RBBP7/RFC2/RFC3/RNASEH2A/RPA3/RRM2/SMC1A/SPAG5/SPC24/SPC25/STMN1/TACC3/TCF19/TIMELESS/TK1/TOP2A/TRIP13/TUBB/TUBG1/UBE2T/UBR7/UNG/WEE1

## Data Availability

The data presented in this study are available on request from the corresponding author.
